# Hepatic BRD4 Is Upregulated in Liver Fibrosis of Various Etiologies and Positively Correlated to Fibrotic Severity

**DOI:** 10.3389/fmed.2021.683506

**Published:** 2021-07-14

**Authors:** Cichun Wu, Da Cheng, Yanghui Peng, Ying Li, Chunyan Fu, Ying Wang, Lei Fu, Shifang Peng, Xin Ni

**Affiliations:** ^1^Department of Infectious Diseases, Xiangya Hospital Central South University, Changsha, China; ^2^Department of Pathology, Xiangya Hospital Central South University, Changsha, China; ^3^National Clinical Research Center for Geriatric Disorders, Xiangya Hospital Central South University, Changsha, China; ^4^International Collaborative Research Center for Medical Metabolomics, Xiangya Hospital Central South University, Changsha, China

**Keywords:** liver fibrosis, BRD4, CXCL6, HBV, human samples

## Abstract

Bromodomain-containing protein 4 (BRD4) has been implicated to play a regulatory role in fibrogenic gene expression in animal models of liver fibrosis. The potential role of BRD4 in liver fibrosis in humans remains unclear. We sought to investigate the expression and cellular localization of BRD4 in fibrotic liver tissues. Human liver tissues were collected from healthy individuals and patients with liver fibrosis of various etiologies. RNA-seq showed that hepatic BRD4 mRNA was elevated in patients with liver fibrosis compared with that in healthy controls. Subsequent multiple manipulations such as western blotting, real-time quantitative polymerase chain reaction, and dual immunofluorescence analysis confirmed the abnormal elevation of the BRD4 expression in liver fibrosis of various etiologies compared to healthy controls. BRD4 expression was positively correlated with the severity of liver fibrosis, and also correlated with the serum levels of aspartate aminotransferase and total bilirubin. Moreover, the expression of C-X-C motif chemokine ligand 6 (CXCL6), a factor interplayed with BRD4, was increased in hepatic tissues of the patients with liver fibrosis. Its expression level was positively correlated with BRD4 level. BRD4 is up-regulated in liver fibrosis, regardless of etiology, and its increased expression is positively correlated with higher degrees of liver fibrosis. Our data indicate that BRD4 play a critical role in the progress of liver fibrosis, and it holds promise as a potential target for intervention of liver fibrosis.

## Introduction

Hepatic fibrosis is a pathological process involving persistent injury to the liver and subsequent wound-healing responses that induce the production and deposition of extracellular matrix (ECM) proteins. Chronic inflammation in response to liver injury and excessive accumulation of ECM proteins can result in the progressive substitution of liver parenchyma by scar tissue. If left untreated, liver fibrosis can progress into cirrhosis, an end stage of liver fibrosis that affects 1–2% of the patient population and causes more than one million deaths yearly across the world (1–3). A variety of etiological factors have been identified, including viral hepatitis infections [e.g., hepatitis B virus (HBV) infection, hepatitis C virus (HCV) infection], excessive alcohol consumption, non-alcoholic steatohepatitis (NASH), autoimmune hepatitis (AIH), and cholestasis. Unfortunately, to date, there is no approved anti-fibrotic medication except for liver transplantation as the last resort of curative therapy for patients with liver cirrhosis. The exact mechanisms underlying the development and progression of hepatic fibrosis remain elusive. It has been documented that the activation of hepatic stellate cells (HSCs) through various inflammatory and fibrogenic pathways plays a pivotal role in liver fibrosis (4, 5). HSCs are considered the primary source of activated myofibroblasts and the cells can produce ECM proteins in the liver. Although rapid progress has been made in the basic research on experimental liver fibrosis, accurate non-invasive biomarkers and effective anti-fibrotic drugs are not available in clinical practice. Better understanding of the molecular mechanisms underlying liver fibrosis may provide new therapeutic and diagnostic targets, and in turn may accelerate the development of treatment and diagnosis for liver fibrosis.

The bromodomain and extra-terminal domain (BET) family is a class of proteins with two bromodomains and one extra-terminal domain, which can recognize acetylated lysine in histones (6, 7). There are four types of BET family members in humans: BRD2, BRD3, BRD4, and BRDT. Among them, BRD4 has recently received great interest because it seems to play a critical role in fibrosis. BRD4 is a transcriptional enhancer with two bromodomains. By binding to acetylated chromatin in interphase and mitosis, it acts as a co-activator in the transcription of various genes to modulate the cell cycle. Previous studies in cell culture systems and animal models have indicated that BRD4 promotes the gene expression of collagen type I alpha 1 chain (Col1A1) in response to transforming growth factor (TGF)-β, thereby resulting in fibrosis in the lung (8–10) and liver organs (11, 12). It has been reported that knockout of BRD4 through its specific small interfering RNA (siRNA) or inhibition using the BRD4 inhibitor JQ1 markedly reduces profibrotic mRNA expression, suppresses platelet derived growth factor (PDGF)-mediated proliferation of HSCs, and blocks the activation of HSCs in the carbon tetrachloride (CCl4)-induced mouse model of liver fibrosis (12–14).

Although BRD4 has been implicated in the development and progression of experimental liver fibrosis in cell culture systems and animal models (11, 15), its expression and function in liver fibrosis in humans has not been elucidated. Intrigued by the previous findings, we performed the first study to characterize the expression of BRD4 in patients with liver fibrosis. Specifically, we examined the BRD4 expression pattern in human liver tissues from patients with liver fibrosis of various etiologies, elucidated the specific cell types of BRD4 expression in human liver fibrosis specimens, and then analyzed the correlation of the BRD4 expression level with the fibrotic degree and clinical features in liver fibrosis caused by chronic HBV infection. A number of studies have shown that BRD4 could be a co-activator for transcription of nuclear factor-κB (NF-κB)-regulated cytokines (8, 16–18). More recently, C-X-C Motif Chemokine Ligand 6 (CXCL6) was reported to be involved in liver fibrosis (19). We therefore examined CXCL6 expression in fibrotic liver tissue and analyzed the correlation between BRD4 and CXCL6 levels. The results showed that BRD4 was highly expressed in the fibrotic liver tissue of patients and was principally localized in hepatocytes, HSCs, macrophages, and biliary tract cells. The BRD4 mRNA level was positively correlated with the CXCL6 mRNA level in the hepatic tissues of patients with liver fibrosis. Moreover, in HBV liver fibrosis, BRD4 protein expression was correlated with the severity of liver fibrosis. Our findings through conducting this study indicate that BRD4 plays important roles in the development and progression of liver fibrosis in humans.

## Materials and Methods

### Human Liver Tissues

A total of 32 fresh liver tissues, comprising 19 samples of liver fibrosis and 13 normal liver tissues, and 96 liver sections were obtained from Xiangya Hospital (Changsha, Hunan, China). Fresh hepatic tissues were collected from the study participants during either surgery or needle biopsy. The normal liver tissues and sections were obtained from transplant donors or individuals with hepatic hemangioma, while fibrotic tissues were obtained from patients with different degrees of liver fibrosis. Immediately following needle biopsy or surgery, fresh liver tissues were collected and snap-frozen in liquid nitrogen or fixed in 4% acetic paraformaldehyde. The liver sections were obtained from patients at Xiangya Hospital spanning the period between January 2015 and January 2020. The 96 liver sections evaluated consisted of 10 samples in the normal control group and 86 samples in the liver fibrosis/cirrhosis group. Liver sections in the normal control group were obtained from benign lesions such as hepatic hemangioma. The liver fibrosis/cirrhosis group contained 40 patients with HBV-associated fibrosis/cirrhosis, three with HCV-associated fibrosis/cirrhosis, one with NASH-associated fibrosis, 13 with cholestasis, five with primary sclerosing cholangitis (PBC), 12 with AIH, and 12 with overlap syndromes (Supplementary Tables 1, 2). The HBV liver fibrosis/cirrhosis samples were classified as F1-F4 according to the Metavir score: F1, portal fibrosis; F2, fibrosis with few septa; F3, numerous septa; and F4, cirrhosis. The clinical characteristics of the enrolled patients are listed in Supplementary Table 3.

Collections of tissues were performed with the approval of the Ethics Review Board of Xiangya Hospital Central South University [IRB(S) No.201912533]. Informed consent was obtained from all patients.

### RNA-Sequencing

Ten snap-frozen liver tissues were used for RNA sequencing (RNA-seq) and were obtained from eight patients who underwent surgical resection for the treatment of HBV-associated liver cancer in the liver fibrosis group (*n* = 4) or hemangioma in the control group (*n* = 4) (GEO: GSE171294). RNA-seq analysis was performed to explore potential genes related to liver fibrosis (Novogene Bioinformatics Institute, Beijing, China). Briefly, the total RNA was extracted using the RNeasy Mini Kit (QIAGEN, Germantown, USA) following the manufacturer's manual. The NEBNext® UltraTM RNA Library Prep Kit for Illumina® (NEB, USA) was used to generate sequencing libraries according to the manufacturer's instructions. Index codes were added to attribute sequences to each sample, and the index-coded samples were clustered using the TruSeq PE Cluster Kit v3-cBot-HS (Illumina) on a cBot Cluster Generation System in accordance with the manufacturer's manual. Upon the completion of cluster generation, RNA-seq was performed and 150 bp paired-end reads were generated with an Illumina Novaseq platform.

### Quantitative Real-Time Polymerase Chain Reaction

Total RNA was extracted using the TRIzol reagent (Invitrogen, Grand Island, NY) following the manufacturer's instructions. RNA samples (2 μg) were reverse transcribed in cDNA with a reverse transcriptase (Promega, Madison, WI). To determine the mRNA expression levels of target genes, quantitative real-time PCR was performed using the 2 × Taq PCR Master Mix (Qiagen, Beijing, China) and primers (Supplementary Table 4) on the ABI PRISM 7500 Sequence Detection System (Applied Biosystems, Foster City, CA, USA) according to the manufacturer's protocol. Distilled water was used in place of cDNA as a negative control. The relative mRNA expression of the gene of interest was determined using the comparative Ct (threshold cycle) method as reported previously (20). Specifically, the ΔCt in each group was generated by subtracting the Ct of glyceraldehyde 3-phosphate dehydrogenase (GAPDH) from that of the target gene, yielding the ^Δ^Ct in each group. The ^ΔΔ^Ct was then obtained by subtracting the ^Δ^Ct of the experimental group from that of the control group. The relative expression levels of the gene of interest were normalized to those of GAPDH.

### Western Blot Analysis

Total proteins were extracted from each liver tissue sample (50 mg) using a radioimmunoprecipitation assay (RIPA) lysis buffer containing a protease inhibitor cocktail tablet (Roche, Indianapolis, IN), and a bicinchoninic acid (BCA) assay was conducted to determine the protein concentrations. For Western blot analysis, 30 μg of each protein sample was separated by 10% sodium dodecyl sulfate polyacrylamide gel electrophoresis (SDS-PAGE). After completion, all proteins were transferred to nitrocellulose membranes and blocked by incubation with 5% skim milk powder dissolved in 0.1% Tris-buffered saline/Tween 20 (TBST). The resulting membranes were sequentially incubated with primary antibodies, including anti-BRD4 antibody (1:1000, ab128874; Abcam) and anti-GAPDH antibody (1:1,000; ab181602; Abcam) at 4°C overnight. The membranes were subsequently incubated with a secondary horseradish peroxidase-conjugated antibody (Proteintech Inc, Wu Han, China) at room temperature for 1 h. Immunoreactive proteins were visualized using the Enhanced Chemiluminescence Western Blotting Detection System (Santa Cruz) and the intensity of the chemiluminescent signal was analyzed using image J software. To control for sample loading error, the expression of BRD4 was normalized to GAPDH.

### Histological Examinations and Laboratory Tests

Paraffin-embedded sections at a thickness of 2 μm were stained with Masson trichrome for histological examinations of the liver tissues. The pathological changes in the liver tissues were assessed by pathologists at Xiangya Hospital Central South University, and the degrees of liver fibrosis were evaluated according to the Metavir score. Blood routine tests and biochemical examinations for liver function and coagulation function were performed at the clinical lab in Xiangya Hospital Central South University.

### Immunohistochemistry

Immunocytochemistry (IHC) was performed as described previously (21). In brief, paraffin sections (2 μm in thickness) were prepared from paraffin embedded liver tissue blocks, deparaffinized, and subsequently hydrated. Endogenous peroxides were then quenched with 0.3% H2O2. The sections were sequentially incubated with primary antibodies, including anti-BRD4 antibody (ab128874; Abcam, Cambridge, UK) at a dilution of 1:200 and anti-CXCL6 antibody (SC-377026, Santa Cruz) at a dilution of 1:100 at 4°C overnight. Sections with the primary antibody substituted with an equal concentration of rabbit or mouse IgG (ab172730, Abcam) were used as negative controls The bound antibodies were detected with diaminobenzidine (Sigma-Aldrich, St. Louis, MO) using the biotin–streptavidin–peroxidase system (UltraSensitive-SP-kit, MaiXin Biotechnology, Fuzhou, China). A nuclear counterstain with hematoxylin was performed in IHC.

During data analysis, both the intensity and extent of staining were taken into consideration. The intensity of staining was determined using the following criteria: 0 for negative; 1 for weak staining; 2 for moderate staining; and 3 for strong staining. The extent of staining was expressed as the area percentage of positive staining, ranging from 0 to 100%. Then, 10 areas from each section were randomly selected to examine the area percentage of positive staining and to calculate the mean extent of staining. The final score for each section was obtained by multiplying these two scores (intensity score × extent score) as reported previously (21).

### Immunofluorescence

Paraffin sections at a thickness of 2 μm were rehydrated, after which the slides were incubated with sodium citrate buffer (pH 6.0) for 10 min to retrieve the antigens. The slides were incubated for 1 h with 10% (v/v) bovine serum albumin (BSA), a widely used blocking solution, to minimize the background and non-specific binding. The resulting slides were sequentially incubated with primary antibodies at 4°C overnight, specifically anti-BRD4 antibody (ab128874, Abcam), anti-hepatocyte nuclear factor (HNF)4α antibody (ab41898; Abcam), anti-α-smooth muscle actin (SMA) antibody (ab7817; Abcam), anti-cluster of differentiation (CD)68 antibody (ab201340; Abcam), and anti-cytokeratin (CK)19 antibody (ab194399; Abcam). The resulting slides were washed three times with PBS, followed by incubation with secondary antibodies labeled with either Coralite Fluor 488 or Coralite Fluor 594. These procedures were performed in accordance with the manufacturer's instructions. The nuclei were stained with 4′,6-diamidino-2-phenylindole (DAPI). The slides were then mounted with cover glass, visualized, and analyzed under a Zeiss LSM 780 confocal microscope (Carl Zeiss Inc., Jena, Germany).

### Statistical Analysis

Statistical analysis was conducted using the SPSS version 20 software package. Measurement data were analyzed with the Shapiro-Wilk test for normality, and data with a normal distribution were expressed as mean ± SEM. Differences between groups were evaluated using two independent samples *t*-test. Data with a non-normal distribution were presented as the median M (P25, P75) and differences between groups were assessed with the Mann-Whitney U rank-sum test. Count data were presented as the number of cases (percentage) and differences between groups were analyzed with the χ2 test. Spearman's correlation and Pearson correlation were used for the correlation analysis. *P*-values < 0.05 were considered to indicate a significant difference between groups.

## Results

### Hepatic BRD4 mRNA and Protein Expression Was Up-Regulated in Liver Fibrosis and Correlated to CXCL6 Level

**Figure 1:**
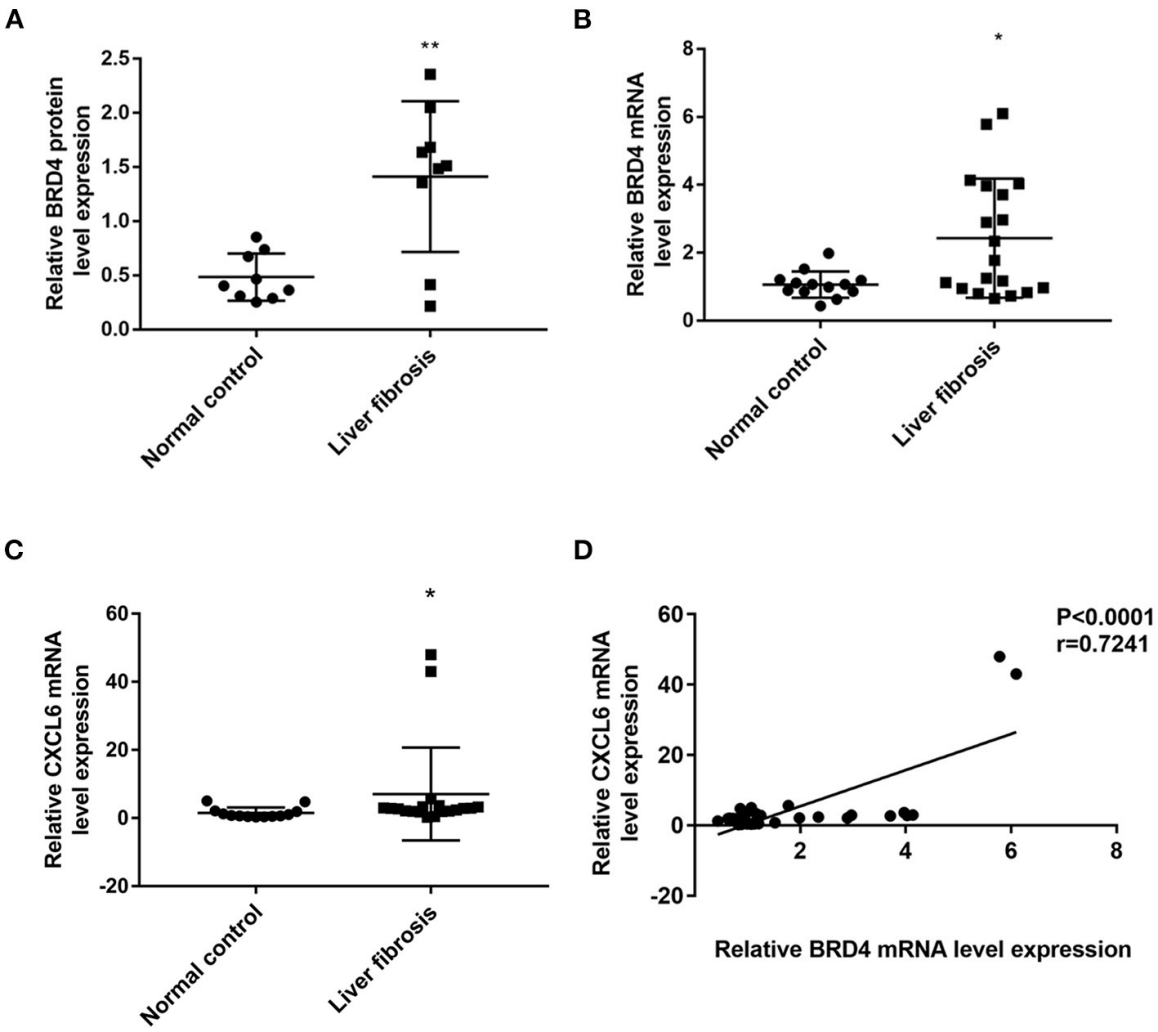
BRD4 and CXCL6 expression in liver tissues of control and fibrosis group. The hepatic tissues were obtained as described in Materials and Methods. **(A)** BRD4 protein expression in patients with liver fibrosis (*n* = 9) and normal controls (*n* = 9). Data were expressed as mean ± SEM, Differences between groups were evaluated using two independent samples *t*-test. **(B)** mRNA expression level of BRD4 in liver tissues from patients with liver fibrosis (*n* = 19) and normal controls (*n* = 13). Data were expressed as median M (P25, P75), Differences between groups were evaluated using Mann-Whitney U rank-sum test. **(C)** CXCL6 mRNA expression in liver fibrosis group (*n* = 19) and control group (*n* = 13). Data were expressed as median M (P25, P75). Differences between groups were assessed using Mann-Whitney U rank-sum test. **(D)** Spearman correlation analysis of correlation between CXCL6 mRNA and BRD4 mRNA expression. **P < 0.01, *P < 0.05 vs. control. BRD4, Bromodomain-containing protein 4; CXCL6, C-X-C motif chemokine ligand 6.

RNA-seq showed that 1,246 genes were aberrantly expressed with more than log2-fold change in liver fibrosis tissues than in normal control tissues (*P* < 0.05). Among these, 687 genes were significantly up-regulated and 559 genes were down-regulated (Supplementary Figure 1). Notably, the BRD4 gene was increased in the liver fibrosis group vs. the normal control group (Supplementary Figure 2). In a relatively large scale of liver biopsy samples, qRT-PCR showed that BRD4 mRNA expression was significantly up-regulated in the fibrosis group compared with the expression in the control group (Figure 1B). Western blot analysis also showed that the BRD4 protein level was significantly increased in the fibrosis group compared with the level in the control group (Figure 1A and Supplementary Figure 3).

It was recently reported that hepatic CXCL6 expression is up-regulated in liver fibrosis and can promote the interaction of BRD4 with other transcriptional factors (19). RNA-seq showed that CXCL6 mRNA expression was increased in fibrotic liver tissues (Supplementary Figure 2). Confirmatory qRT-PCR analysis showed that the CXCL6 mRNA level was significantly increased in liver tissues from the liver fibrosis group compared with those from the control group (Figure 1C). Interestingly, the CXCL6 mRNA level was positively correlated to the BRD4 mRNA level in the liver fibrosis group (Figure 1D). Immunohistochemistry showed that CXCL6 positive staining was mainly localized in hepatocytes in liver tissues (Supplementary Figure 4).

### Hepatic BRD4 Expression Was Increased in Liver Fibrosis/Cirrhosis With Various Etiological Factors

**Figure 2:**
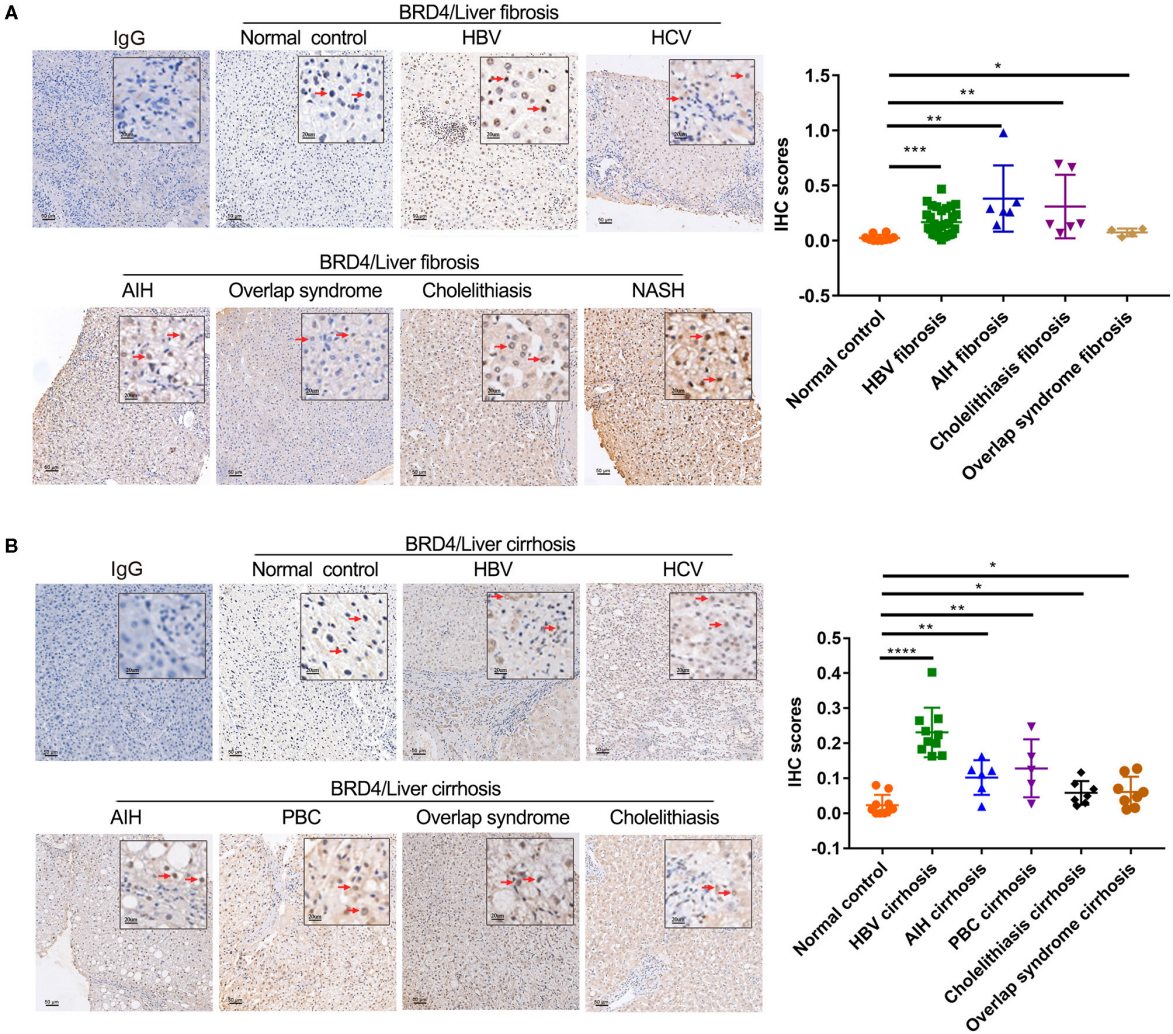
Immunohistochemistry analysis of hepatic BRD4 protein expression in liver fibrosis/cirrhosis. **(A)** Immunohistochemistry analysis was performed to examine hepatic BRD4 protein expression patterns in normal liver tissues and liver fibrosis of various etiologies. The control sections were incubated with IgG. Representative images of normal hepatic tissues, HBV, HCV, AIH, NASH, cholelithiasis, and overlap syndrome. Scale bar: 50 μm. The expression level of BRD4 was analysis by Image J software. Data were expressed as mean ± SEM or median M (P25, P75). Differences between groups were evaluated using two independent samples *t*-test or Mann-Whitney U rank-sum test. **(B)** Immunohistochemistry analysis was performed to examine hepatic BRD4 protein expression patterns in normal liver tissues and liver cirrhosis of various etiologies. The control sections were incubated with IgG. Representative images of normal hepatic tissues, HBV, HCV, AIH, PBC, cholelithiasis, and overlap syndrome. Scale bar: 50 μm. The expression level of BRD4 was analysis by Image J software. Data were expressed as mean ± SEM or median M (P25, P75). Differences between groups were evaluated using two independent samples *t*-test or Mann-Whitney U rank-sum test. *P < 0.05; **P < 0.01; ***P < 0.0001; ****P < 0.00001 vs. normal control. BRD4, bromodomain-containing protein 4; HBV, hepatitis B virus; HCV, hepatitis C virus; AIH, autoimmune hepatitis; NASH, non-alcoholic steatohepatitis; PBC, primary sclerosing cholangitis.

Considering that fresh liver samples were limited to liver fibrosis/cirrhosis samples in the vicinity of liver cancer, we examined the hepatic BRD4 expression pattern using pathologic specimens from patients with liver fibrosis/cirrhosis with a variety of etiologies, including HBV, HCV, AIH, PBC, NASH, cholestasis, and overlap syndrome. The analysis showed that BRD4 was mainly expressed in hepatocytes in the liver (Figures 2A,B). A low amount of positive staining for BRD4 was found in fibrotic scars in the liver. BRD4 protein expression was increased in the samples from all enrolled patients with liver fibrosis/cirrhosis compared with the expression in the control samples. As shown in Figures 2A,B, BRD4 protein levels were significantly increased in HBV-, AIH-, PBC-, cholestasis-, and overlap syndrome-associated liver fibrosis/cirrhosis tissues.

### BRD4 Expression Was Localized in Hepatocytes, HSCs, Hepatic Macrophages, and Biliary Tract Cells

**Figure 3:**
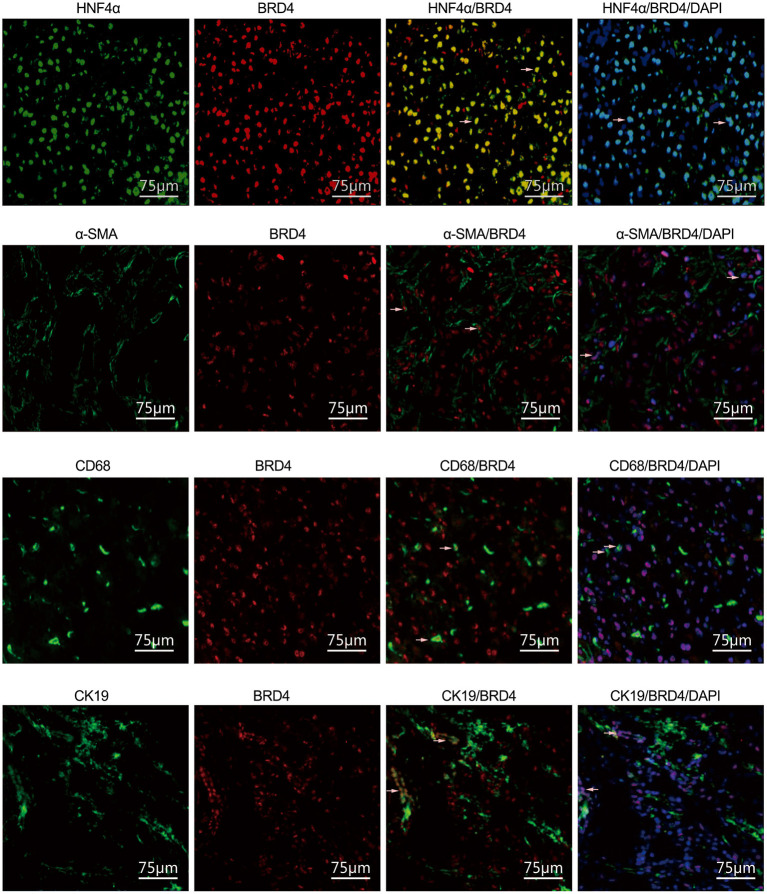
Immunofluorescence analysis of cellular localization of BRD4 in the liver tissues. Immunofluorescence (IF) analysis was conducted to visualize the cellular localization of BRD4 in the liver tissues as described in Materials and Methods. The representative double immunofluorescence images showed expression of HNF4α (green), α-SMA (green), CD68 (green), CK19 (green), and BRD4 (red) in normal and HBV fibrotic liver tissue. Scale bar: 75 μm. BRD4, bromodomain-containing protein 4; HNF4α, hepatocyte nuclear factor 4 alpha; α-SMA, α-smooth muscle actin; CD68, cluster of differentiation 68; CK19, cytokeratin 19.

We further analyzed the cellular localization of BRD4 expression in the cell types of the liver. HNF4α, α-SMA, CD68, and CK19 are cell markers of hepatocytes, activated HSCs, macrophages, and biliary tract cells, respectively. BRD4-positive staining was detected in hepatocytes, activated HSCs, macrophages, and biliary tract cells (Figure 3). It was found that ~ 90% of the total hepatocytes, 56% of the total macrophages and 67% of the total biliary tract cells were BRD4 positive.

### BRD4 Expression Was Positively Correlated With the Severity of Fibrosis and Circulatory Aspartate Aminotransferase and Total Bilirubin Levels in HBV-Induced Liver Fibrosis

Given that HBV-induced liver fibrosis is the most common type of liver fibrosis in China due to the high prevalence of HBV infection, we further analyzed the association of the BRD4 expression level with fibrosis severity and other parameters. The male to female ratio was 1/1 in the normal control group, 1/1 in the F1 group, 4/1 in the F2 group, 9/1 in the F3 group, and 4/1 in the F4 group. No statistically significant difference in age was found among the five subgroups. The age was 51.4 ± 10.6 in the normal control group, 45.1 ± 13.4 in the F1 group, 54.8 ± 12.1 in the F2 group, 48.3 ± 12.9 in the F3 group, and 49.4 ± 14.0 in the F4 group. No statistically significant differences in gender and age were found among the five subgroups (Supplementary Table 3). The clinicopathological liver samples with HBV-associated fibrosis/cirrhosis were graded according to the Metavir score (F1–F4) (Supplementary Table 3). As shown in Supplementary Table 3, the alanine aminotransferase (ALT) levels of the normal control group [11.6 (9.8, 29.9) U/L] were significantly different from those of the F2 group [43.8 (27.0, 58.6) U/L], F3 group [53.3 (23.5, 76.9) U/L], and F4 group [33.5 (24.6, 49.3) U/L] (*P* < 0.05). The AST levels of the normal control group [17.8 (15.1, 28.6) U/L] were also significantly different from those of the F2 group [40.4 (31.5, 60.4) U/L], F3 group [52.7 (27.9, 74.6) U/L], and F4 group [40.4 (29.3, 45.8) U/L] (P < 0.05). The TBIL levels of the normal control group (8.5 ± 2.2 umol/L) were significantly different from those of the F3 group (17.2 ± 6.7 umol/L), F4 group [14.3 (12.1, 17.8) umol/L] (*P* < 0.05). The white blood cell (WBC) level for the control group was significantly different from that of the F3 group (*P* < 0.05). Significant differences were observed between the control group and F4 group in ALT, AST, TBIL, prothrombin time (PT), plasma thromboplastin antecedent (PTA), international normalized ratio (INR), and platelet count (PLT) (*P* < 0.05).

**Figure 4:**
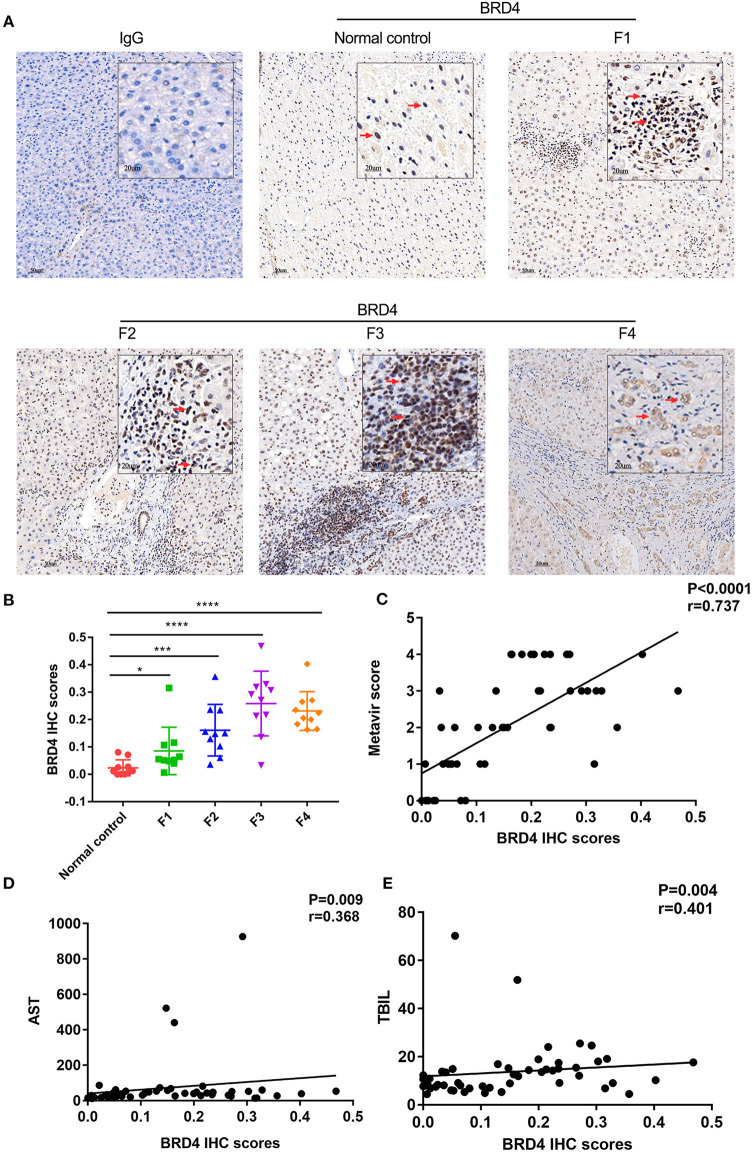
BRD4 protein expression level in hepatic tissues of HBV liver fibrosis with different degrees. Immunohistochemistry was performed to examine hepatic BRD4 protein expression in HBV liver fibrosis with F1–F4 according to the Metavir score. The expression level of BRD4 was analysis by Image J software. **(A)** representative images of BRD4 expression in F1–F4 liver fibrosis. Scale bar: 50 μm. **(B)** cumulative data of BRD4 expression in F1–F4 liver tissues. Data were expressed as mean ± SEM or median M (P25, P75). Differences between groups were evaluated using two independent samples *t*-test or Mann-Whitney U rank-sum test. **(C)** Spearman correlation analysis of the correlation between severity of liver fibrosis and BRD4 expression. **(D)** Spearman correlation analysis was conducted to assess the correlation between BRD4 expression and AST of patients with liver fibrosis. **(E)** Spearman correlation analysis was conducted to assess the correlation between BRD4 expression and TBIL of patients with liver fibrosis. BRD4, bromodomain-containing protein 4; HBV, hepatitis B virus; AST, aspartate aminotransferase; TBIL, total bilirubin. *P < 0.05; ***P < 0.0001; ****P < 0.00001.

As shown in Figures 4A,B, the BRD4 positive staining was increased with the severity of fibrosis. The positive correlation was found between BRD4 level and fibrotic severity (*r* = 0.737, *P* < 0.0001, *n* = 50) (Figure 4C). The BRD4 protein expression was also positively correlated with AST (*r* = 0.368, *P* = 0.009, *n* = 50), TBIL (*r* = 0.401, *P* = 0.004, *n* = 50) (Figures 4D,E). No significant correlation was found between the expression level of BRD4 and the ALT, PT, PTA, INR, or other clinical parameters (Supplementary Figure 5).

## Discussion

The acetyl-histone binding protein BRD4 is not only involved in cell transcription, proliferation, and apoptosis (22–24), but is also essential for the transcription of aurora B kinase, an important kinase in the modulation of chromosome separation and cytokinesis during mitosis (25). More recently, it has been shown that the inhibition of BRD4 activity by its selective inhibitors displays therapeutic potential for liver cancer, gastric cancer, pituitary adenoma, aeroallergen-induced inflammation, and airway remodeling (26–28). Emerging evidence suggests that BRD4 is involved in the development and progression of fibrosis in different organs, including cardiac (29–31), pulmonary (8), and hepatic fibroses (12). The BRD4 selective inhibitor JQ1 has been demonstrated to reverse the fibrosis response in the mouse model of liver fibrosis caused by CCl4 (12). In this study, we obtained the following major novel findings: (1) Hepatic BRD4 expression was up-regulated in patients with liver fibrosis of various etiologies, including HBV, HCV, AIH, PBC, cholestasis, and overlap syndrome; (2) Hepatic BRD4 expression was positively correlated with the severity of liver fibrosis; (3) Increased expression levels of hepatic BRD4 were positively correlated with the circulatory parameters associated with hepatic functions in HBV-induced liver fibrosis. Thus, our data suggest that the up-regulation of BRD4 may be associated with a common pathway for liver fibrosis, rather than the cause of liver fibrosis.

Previous studies have mainly focused on the effects of BRD4 on HSCs and have shown that the up-regulation of BRD4 in HSCs promotes the production of collagen I and subsequently leads to the activation of HSCs (32). However, the cell types that specifically express BRD4 have not been characterized in human liver tissues. In the present study, we demonstrated that BRD4 is expressed in various cell populations in the liver, including not only HSCs, but also hepatocytes, macrophages, and biliary tract cells. However, the functions of BRD4 in these cells in the liver remain largely unknown. It is well-known that the destruction and regeneration of hepatocytes occurs during the development of liver fibrosis caused by HBV. Our results showed that BRD4 protein expression was positively correlated with AST and TBIL in HBV-induced liver fibrosis. In view of the role of BRD4 in cell proliferation and apoptosis, BRD4 might be involved in the regeneration of hepatocytes in HBV-induced liver fibrosis. Some studies have demonstrated that BRD4 plays important roles in macrophages. For instance, a number of recent studies have shown that BRD4 can affect tumor-associated macrophages (TAM) in solid tumors (18, 33, 34). Ren et al. (13) reported that BRD4 participates in the production of pro-inflammatory cytokines induced by titanium particles via promoting the activation of NF-κB signaling in macrophages in mice. Macrophages have recently been shown to play a critical role in liver fibrosis in NASH (35), suggesting that the up-regulation of BRD4 in macrophages might be involved in the progression of liver fibrosis. Nevertheless, the roles of BRD4 in hepatocytes, macrophages, and biliary tract cells during liver fibrosis remain to be further studied.

It is currently recognized that inflammatory factors promote the activation of HSCs and play an important role in the development of liver fibrosis. In various tissues, BRD4 is found to be involved in inflammatory responses. It has been shown that BRD4 plays an important role in inflammation of vascular smooth muscle cells (36). BRD4 inhibitors block airway inflammation caused by the virus (37). BRD4 blockers reduce pathological cardiac hypertrophy by reducing ROS production and inhibiting fibrosis and inflammation (38). BRD4 inhibitors also have a protective effect against vincristine induced peripheral neuropathy by reducing inflammation (39). It has been demonstrated that involvement of BRD4 in inflammation is associated with NF-κB activation. BRD4 plays an central role in respiratory syncytial virus-induced inflammation and infant pneumonia through NF-κB (17, 40). BRD4 inhibitors can effectively inhibit NF-κB mediated inflammatory responses in endothelial cells (41).

Given that BRD4 is a co-activator of NF-κB and promotes the transcription of chemokines and cytokines in various cells besides macrophages, there is a possibility that BRD4 may promote the initiation, development, and progression of liver fibrosis through certain inflammatory mediators. It has been shown that chemokines, a group of small (8–14 kDa) proteins, exert their roles in the host immune responses and other physiological and pathological processes through interactions with their cell-surface receptors (42–45). Chemokines are produced by a wide range of cell types, including leukocytes, endothelial cells, fibroblasts, epithelial cells, and tumor cells (46–51). Notably, a number of previous studies have shown that the CXCL6 level is increased in the sera of patients with liver fibrosis and its expression is increased in the liver tissues of patients with liver fibrosis (19, 52, 53). It has been reported that CXCL6 is a prognostic biomarker for liver fibrosis. Consistent with these previous studies, we also found that CXCL6 expression is elevated in HBV-induced liver fibrosis. As for the potential role of CXCL6 in liver fibrosis, it has been demonstrated that CXCL6 is involved in the recruitment of monocytes into the liver following liver injury. It stimulates the release of TGF-β by Kupffer cells and subsequently promotes the activation of HSCs, thereby leading to liver fibrosis (54). Interestingly, Cai et al. (19) also showed that CXCL6 could promote interactions between BRD4 and other transcription factors (e.g., SMAD2) in HSCs. These findings are in support of our speculation that BRD4 could promote CXCL6 production through its function as a co-activator of NF-κB, and that CXCL6, in turn, promotes BRD4 function by enhancing interactions between BRD4 and other factors. Such interactions between CXCL6 and BRD4 might promote the development and progression of liver fibrosis.

The present study has some potential limitations. For instance, we did not verify whether CXCL6 is the downstream gene of BRD4 and did not assess the potential genes regulated by BRD4 in human liver tissues. Further in-depth investigations are underway in our laboratory to better understand the role of BRD4 in patients with liver fibrosis.

In summary, this study has demonstrated, for the first time to our knowledge, that hepatic BRD4 expression is markedly increased in patients with liver fibrosis with various etiological factors, and that elevated levels of BRD4 are associated with higher degrees of liver fibrosis. The results have also indicated that BRD4 is mainly expressed in hepatocytes, HSCs, hepatic macrophages, and biliary tract cells in the liver. These findings suggest that BRD4 plays an important role in the development and progression of liver fibrosis in humans and that BRD4 has potential as a target for intervention of liver fibrosis.

## Data Availability Statement

The datasets presented in this study can be found in online repositories. The name of the repository and accession number can be found below: National Center for Biotechnology Information (NCBI) Gene Expression Omnibus (GEO), https://www.ncbi.nlm.nih.gov/geo/, GSE171294.

## Ethics Statement

The studies involving human participants were reviewed and approved by Ethics Review Board of The Xiangya Hospital of Central South University. The patients/participants provided their written informed consent to participate in this study.

## Author Contributions

XN designed the experiments, wrote the manuscript, and supervised the study. SP and LF supervised the study and instructed the clinical characteristics. CW performed the experiments and wrote the manuscript. DC assisted the data analysis and experiments. YP and YL assisted experiments. CF and YW instructed the histological analysis of the section. All authors contributed to the article and approved the submitted version.

## Conflict of Interest

The authors declare that the research was conducted in the absence of any commercial or financial relationships that could be construed as a potential conflict of interest.

## Abbreviations

AIH: autoimmune hepatitis
AST: aspartate aminotransferase
BCA: bicinchoninic acid
BET: bromodomain and extra-terminal domain
BRD4: Bromodomain-containing protein 4
BSA: bovine serum albumin
CCl4: carbon tetrachloride
CD: cluster of differentiation
CK: cytokeratin
Col1A1: collagen type I alpha 1 chain
CXCL6: C-X-C Motif Chemokine Ligand 6
DAPI: 4′6-diamidino-2-phenylindole
ECM: extracellular matrix
HBV: hepatitis B virus
HCV: hepatitis C virus
AIH: autoimmune hepatitis
NASH: non-alcoholic steatohepatitis
PBC: primary sclerosing cholangitis
HNF: hepatocyte nuclear factor
HSCs: hepatic stellate cells
IF: immunofluorescence
IHC: immunocytochemistry
NASH: non-alcoholic steatohepatitis
NF-κB: nuclear factor-κB
PDGF: platelet derived growth factor
qRT-PCR: real-time quantitative polymerase chain reaction
RIPA: radioimmunoprecipitation assay
RNA-seq: RNA sequencing
SDS-PAGE: sodium dodecyl sulfate polyacrylamide gel electrophoresis
siRNA: small interfering RNA
αSMA: α-smooth muscle actin
TBIL: total bilirubin
TBST: Tris-buffered saline/Tween 20
TGF-β: transforming growth factor β
WB: Western blot.
