# Supplementary material for: Hepatic BRD4 Is Upregulated in Liver Fibrosis of Various Etiologies and Positively Correlated to Fibrotic Severity
Source: Front Med (Lausanne). 2021 Jul 14;8:683506. doi: 10.3389/fmed.2021.683506 (PMC8317578; doi:10.3389/fmed.2021.683506)
Supplement: Supplementary file 1 [file Data_Sheet_1.docx]

Supplementary Material

**Supplementary Tables**

**Table S1** Clinical characteristics and biochemical results of healthy control and patients with hepatic fibrosis

|  | Control(n=10) | HBV(n=30) | HCV(n=2) | AIH(n=6) | NASH  (n=1) | Overlap syndromen  (n=4) | Cholelithiasis  (n=6) |
| --- | --- | --- | --- | --- | --- | --- | --- |
| Gender |  |  |  |  |  |  |  |
| Male | 5(50%) | 22(73%) | 1(50%) | 1(17%) | 1(100%) | 0(0%) | 1(17%) |
| Female | 5(50%) | 8(27%) | 1(50%) | 5(83%) | 0(0%) | 4(100%) | 5(83%) |
| Age(years) | 51.4±10.6 | 49.5(36.8,61.5) | 45.0 | 53.8±7.8 | 59.0 | 54.5±11.2 | 56.0±6.4 |
| ALT(u/l) | 11.6(9.8,29.9) | 43.4(23.5,59.0) | 30.4 | 116.9(43.0,518.6) | 77.1 | 87.8±9.1 | 106.3(13.1,115.8) |
| AST(u/l) | 17.8(15.1,28.6) | 43.4(23.5,59.0) | 31.4 | 110.3(43.1,494.1) | 38.8 | 160.0±54.4 | 69.9±36.5 |
| TBIL (umol/l) | 8.5±2.2 | 10.9(7.0,17.1) | 11.1 | 38.8±30.6 | 4.5 | 101.3±84.4 | 21.6(13.9,130.5) |
| PT(s) | 12.5(11.9,14.3) | 13.4(12.9,14.0) | 13.2 | 14.5±1.7 | 12.6 | 12.8±1.1 | 13.3±0.9 |
| PTA (%) | 110.9(91.8,115.6) | 98.9(92.4,108.5) | 103.3 | 89.7±18.7 | 109.8 | 105.8±13.6 | 92.6±16.5 |
| INR | 1.0(1.0,1.1) | 1.1(1.0,1.1) | 1.0 | 1.1±1.1 | 1.0 | 1.0±0.1 | 1.1±0.1 |
| WBC(x10*9/l) | 5.3±1.1 | 5.5(4.7,7.5) | 6.0 | 4.4±1.3 | 6.3 | 5.9±2.1 | 7.5±2.8 |
| PLT(x10*9/l) | 175.4±38.1 | 181.0(137.8,207.3) | 96.0 | 119.0(89.0,175.5) | 124.0 | 195.5(195.0,200.5) | 243.7±53.6 |

Note: Values were expressed as number (percentage) or median M (P25, P75) or mean ± standard deviation. HBV, hepatitis B virus; HCV, hepatitis C virus; AIH, autoimmune hepatitis; NASH, non-alcoholic steatohepatitis.

**Table S2** Clinical characteristics and biochemical results of healthy control and patients with liver cirrhosis

|  | Control(n=10) | HBV(n=10) | HCV(n=1) | AIH(n=6) | PBC(n=5) | Overlap syndrome  (n=8) | Cholelithiasis  (n=7) |
| --- | --- | --- | --- | --- | --- | --- | --- |
| Gender |  |  |  |  |  |  |  |
| Male | 5(5%) | 8(80%) | 1(100%) | 1(17%) | 2(40%) | 1(13%) | 1(14%) |
| Female | 5(50%) | 2(20%) | 0(0%) | 5(83%) | 3(60%) | 7(87%) | 6(86%) |
| Age (years) | 51.4±10.6 | 49.4±14.0 | 61.0 | 59.8±4.8 | 46.6±8.2 | 49.9±11.4 | 50.0(49.0,70.0) |
| ALT (u/l) | 11.6(9.8,29.9) | 33.5(24.6,49.3) | 18.1 | 89.3(67.5,152.4) | 113.2±99.1 | 88.5±48.5 | 62.5±42.5 |
| AST (u/l) | 17.8(15.1,28.6) | 40.4(29.3,45.8) | 30.9 | 122.6(87.4,222.2) | 152.1±128.5 | 124.8±54.6 | 58.8(37.1,78.0) |
| TBIL (umol/l) | 8.5±2.2 | 14.3(12.1,17.8) | 38.1 | 22.0(21.6,54.0) | 58.0(31.2,419.5) | 63.4(34.6,141.4) | 20.6(13.4,130.9) |
| PT (s) | 12.5(11.9,14.3) | 14.1(13.4,14.9) | 15.5 | 15.3(14.5,21.6) | 13.5(13.0,19.4) | 15.0(13.5,22.3) | 13.4(13.0,14.2) |
| PTA (%) | 110.9(91.8,115.6) | 90.8(80.3,95.2) | 70.3 | 69.6±28.1 | 103.0(64.3,105.4) | 69.5±30.5 | 94.0(92.3,103.3) |
| INR | 1.0(1.0,1.1) | 1.1(1.0,1.2) | 1.2 | 1.2(1.1,1.7) | 1.0(1.0,1.5) | 1.2(1.1,1.8) | 1.0(1.0,1.1) |
| WBC (x10*9/l) | 5.3±1.1 | 4.7±2.2 | 5.1 | 4.1(2.8,5.8) | 4.2(4.0,7.4) | 6.8±2.5 | 3.4±1.1 |
| PLT (x10*9/l) | 175.4±38.1 | 126.3±61.8 | 71.0 | 102.7±27.6 | 151.0±52.7 | 124.3±67.4 | 114.1±37.0 |

Note: Values were expressed as number (percentage) or median M (P25, P75) or mean ± standard deviation. HBV, hepatitis B virus; HCV, hepatitis C virus; AIH, autoimmune hepatitis; PBC, primary sclerosing cholangitis.

**Table S3.** Clinical characteristics and biochemical results of healthy control and patients with HBV hepatic fibrosis

|  | Control（n=10） | F1 （n=10） | F2（n=10） | F3（n=10） | F4（n=10） |
| --- | --- | --- | --- | --- | --- |
| Clinical Features |  |  |  |  |  |
| Gender |  |  |  |  |  |
| Male | 5 (50%) | 5 (50%) | 8 (80%) | 9 (90%) | 8 (80%) |
| Female | 5 (50%) | 5 (50%) | 2 (20%) | 1 (10%) | 2 (20%) |
| Age(years) | 51.4±10.6 | 45.1±13.4 | 54.8±12.1 | 54.8±12.1 | 49.4±14.0 |
| Biochemical results |  |  |  |  |  |
| ALT (u/l) | 11.6 (9.8,29.9) | 33.1±21.2 | 43.8 (27.0,58.6)† | 53.3 (23.5,76.9)† | 33.5 (24.6,49.3)† |
| AST (u/l) | 17.8 (15.1,28.6) | 32.2±15.9 | 40.4 (31.5,60.4)† | 52.7 (27.9,74.6)†‡ | 40.4 (29.3,45.8)† |
| TBIL (umol/l) | 8.5±2.2 | 7.0 (5.7,13.9) | 10.7±5.9 | 17.2±6.7†‡ | 14.3 (12.1,17.8)†‡§ |
| PT (s) | 12.5 (11.9,14.3) | 13.4±0.7 | 13.3±0.6 | 13.7 (13.0,14.3) | 14.1 (13.4,14.9)†‡§ |
| PTA (%) | 110.9 (91.8,115.6) | 100±7.8 | 99.8±6.8 | 92.9 (84.3,104.6) | 90.8 (80.3,95.2)†‡§ |
| INR | 1.0 (1.0,1.1) | 1.1±0.1 | 1.0±0.1 | 1.1 (1.0,1.1) | 1.1 (1.0,1.2)†§ |
| WBC (x10*9/l) | 5.3±1.1 | 5.8±1.4 | 4.9 (4.3,9.3) | 7.2±2.3† | 4.7±2.2# |
| PLT (x10*9/l) | 175.4±38.1 | 170.2±55.9 | 190.0 (175.3,203.8) | 169.5±66.7 | 126.3±61.8†§ |

Note: Values were expressed as number (percentage) or median M (P25, P75) or mean ± standard deviation. Measurement data were analyzed with the Shapiro-Wilk test for normality, and data with a normal distribution were expressed as mean± SEM. Differences between groups were evaluated using two independent samples t-test. Data with a non-normal distribution were presented as the median M (P25, P75) and differences between groups were assessed with the Mann-Whitney U rank-sum test. Count data were presented as the number of cases (percentage) and differences between groups were analyzed with the χ2 test. †. Compared with control group, P<0.05; ‡. Compared with F1 group, P<0.05; §. Compared with F2 group, P<0.05. #. Compared with F3 group, P<0.05. The severity of liver fibrosis was divided into F1-F4 four stages according to the Metavir score. HBV, hepatitis B virus.

**Table S4.** The sequences of primers used for RT-PCR

| GAPDH | Forward: TGTTGCCATCAATGACCCCTT  Reverse: CTCCACGACGTACTCAGCG |
| --- | --- |
| BRD4 | Forward: CTCCCCGCTTATGATACATTCC  Reverse: GTTTCTTAGGCTGGACGTTTTG |
| CXLC6 | Forward: ACGCTGAGAGTAAACCCCAAAACG  Reverse: CGGGTCCAGACAAACTTGCTTCC |

Note: BRD4, bromodomain-containing protein 4; CXCL6, C-X-C motif chemokine ligand 6.

## Supplementary Figures


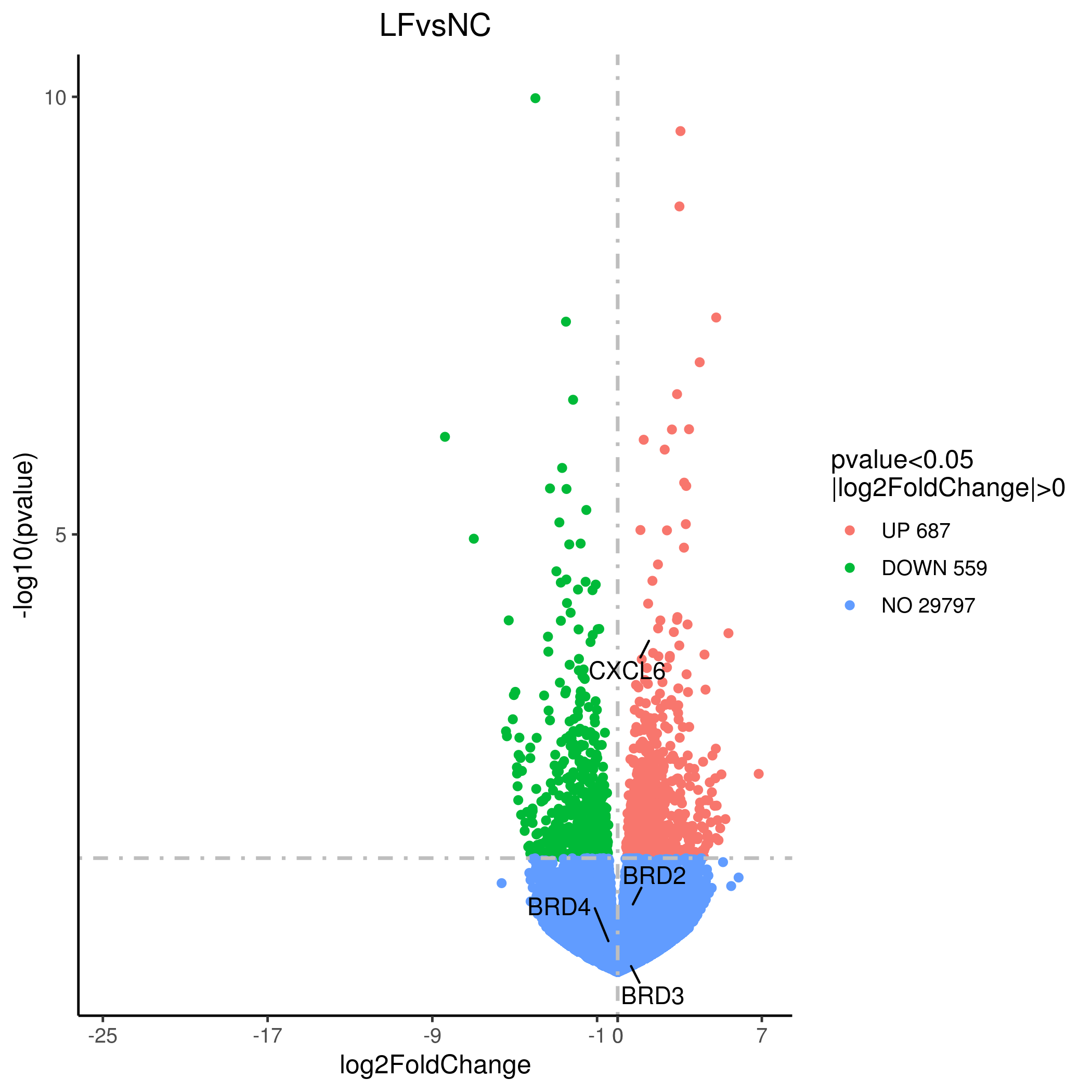


**Figur****e S1 Volcano plot of RNA-sequencing data for analysis of differential expression of hepatic mRNAs in liver fibrosis.** RNA-sequencing (RNA-seq) was conducted using the RNA samples extracted from patients with liver fibrosis and normal controls (n=4 in each group).1240 genes were changed with greater than log2-fold change in liver fibrosis versus normal control. Among them, 687 genes were up-regulated and 559 genes were down-regulated. NC, normal control; LF, liver fibrosis.


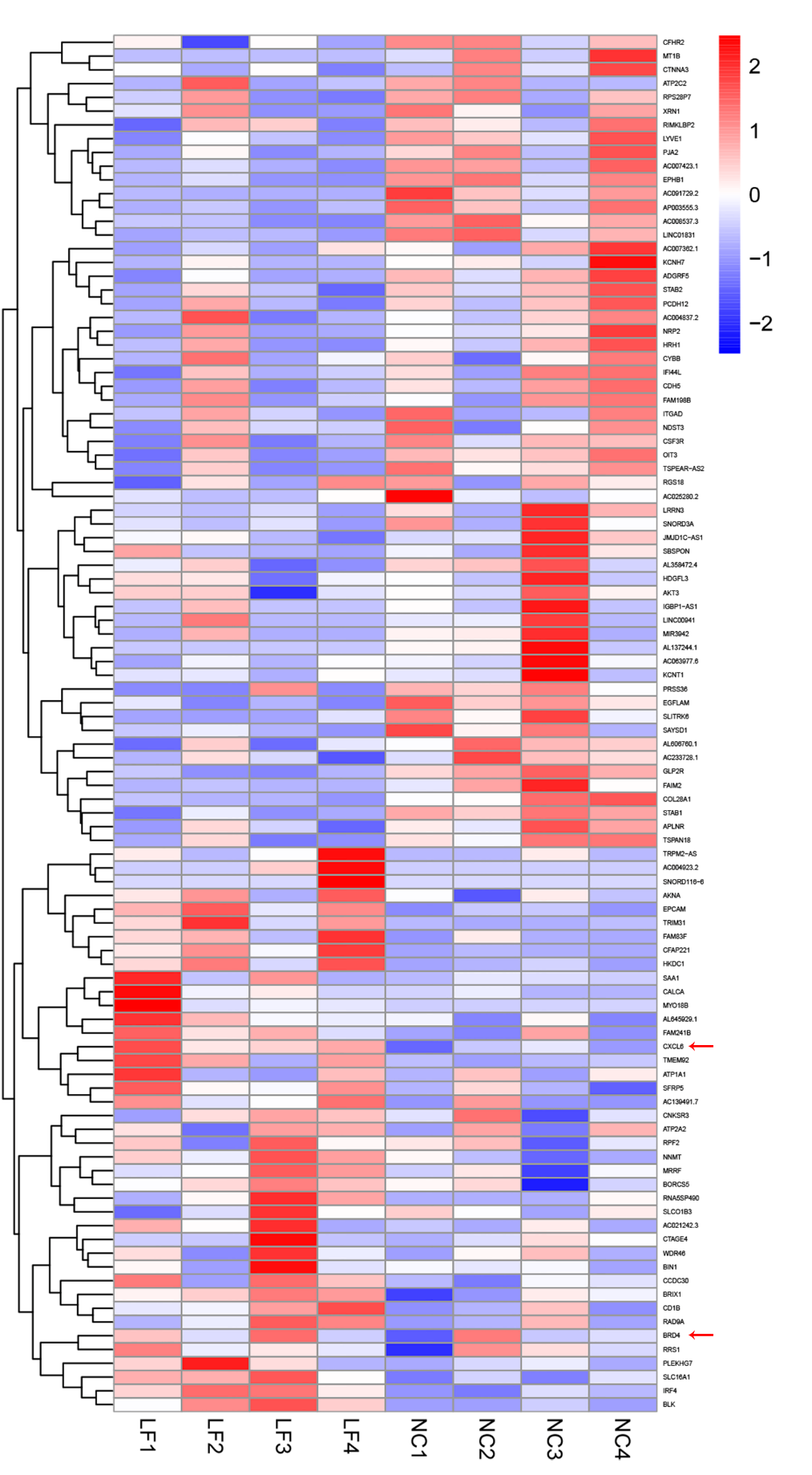


**Figure S2. Clustered heat map of RNA-seq data for analysis of differential exp****ression of hepatic mRNAs in liver fibrosis.** A heat map was constructed using the RNA-seq data. NC, normal control; LF, liver fibrosis.


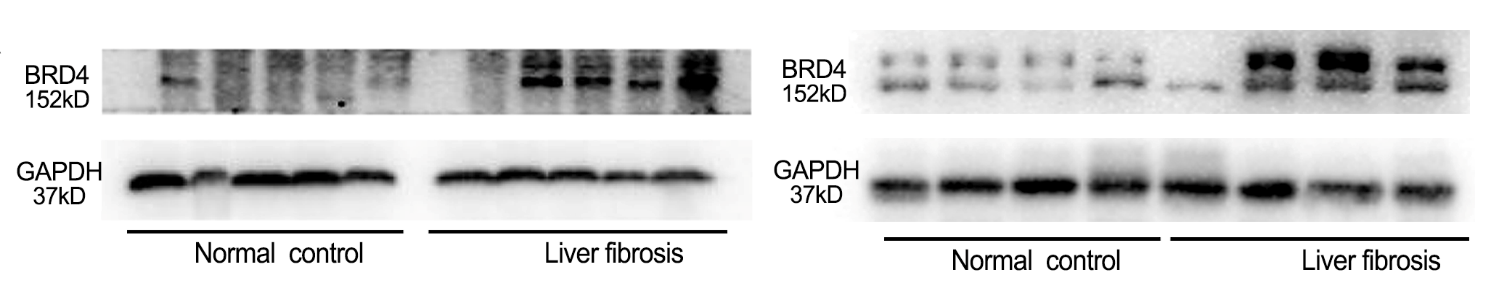


**Figure S3.** **Western blotting images of BRD4 in human hepatic tissues.** The hepatic tissues were obtained as described in Materials and Methods**.** Western Bolt was performed to examine BRD4 protein expression in patients with liver fibrosis（n=9） and normal controls（n=9）.  BRD4, bromodomain-containing protein.


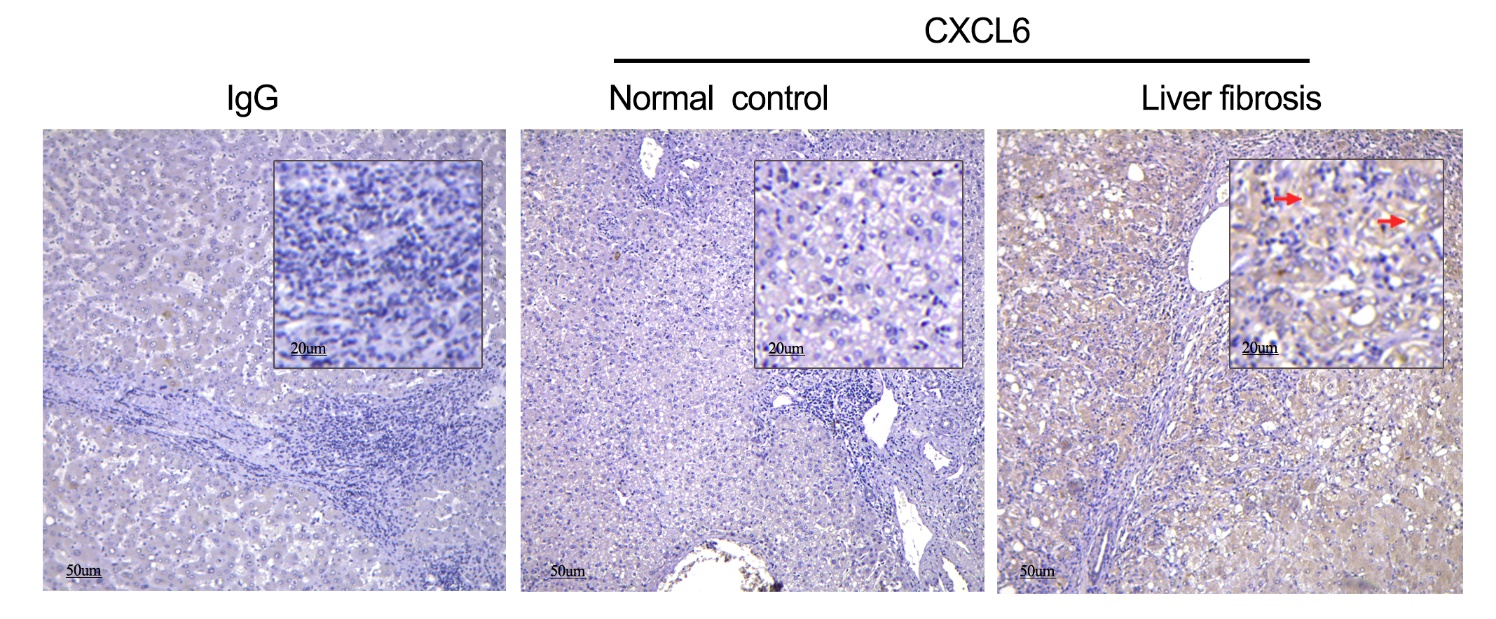


**Figure S4. CXCL6 localization in human hepatic tissues.** Immunohistochemistry analysis was performed to examine hepatic CXCL6 protein expression in liver fibrosis. Arrows indicate the positive staining of CXCL6. Scale bar:50μm. CXCL6, C-X-C motif chemokine ligand 6.


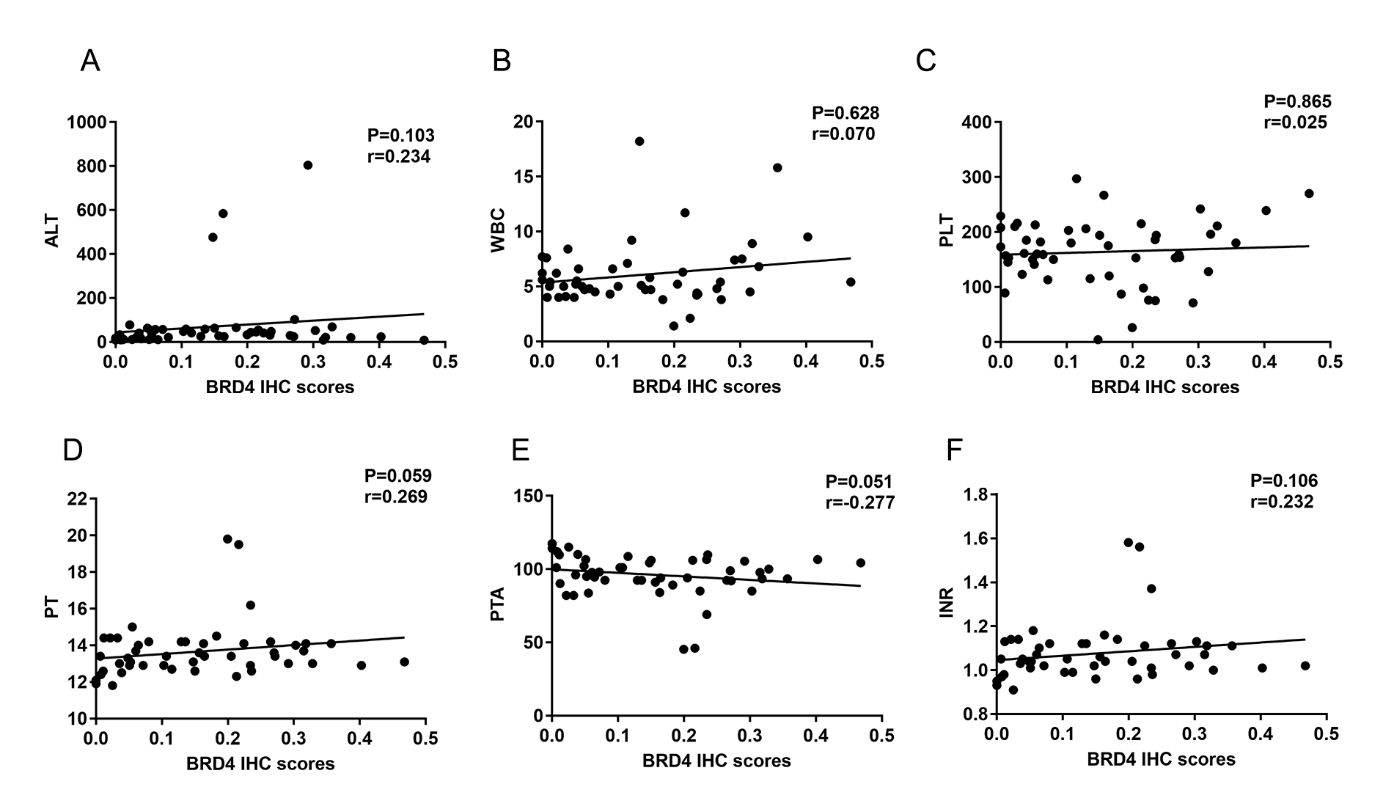
**Figure** **S5. Spearman correlation analysis of the relationship between clinical characteristics and BRD4 expression in liver fibrosis.** Spearman correlation analysis was conducted to assess the correlation between BRD4 expression and clinical characteristics of patients with liver fibrosis. BRD4, bromodomain-containing protein 4; IHC, immunohistochemistry; ALT: alanine aminotransferase;WBC, white blood cell; PLT, platelet count; PT: prothrombin time; PTA, plasma thromboplastin antecedent; INR, international normalized ratio.

.
